# The activity of the saponin ginsenoside Rh2 is enhanced by the interaction with membrane sphingomyelin but depressed by cholesterol

**DOI:** 10.1038/s41598-019-43674-w

**Published:** 2019-05-13

**Authors:** Sandrine L. Verstraeten, Magali Deleu, Maria Janikowska-Sagan, Emily J. S. Claereboudt, Laurence Lins, Donatienne Tyteca, Marie-Paule Mingeot-Leclercq

**Affiliations:** 1 Université catholique de Louvain, Louvain Drug Research Institute, Cellular & Molecular Pharmacology Unit, 1200 Brussels, Belgium; 2 Université catholique de Louvain, de Duve Institute, Cell Biology Unit, 1200 Brussels, Belgium; 3 Université de Liège (ULiège), Gembloux-Agro-Bio Tech, Molecular Biophysics at Interfaces Unit, 5030 Gembloux, Belgium; 4 Jagiellonian University, Faculty of Physics, Astronomy and Applied Computer Science, 30-348, Krakow, Poland

**Keywords:** Membrane biophysics, Molecular medicine

## Abstract

The membrane activity of some saponins, such as digitonin or alpha-hederin, is usually attributed to their interaction with membrane cholesterol (Chol). This contrasts with our recent publication showing that Chol, contrary to sphingomyelin (SM), can delay the cytotoxicity of the saponin ginsenoside Rh2, challenging the usual view that most saponins mediate their membrane effects through interaction with Chol. The aim of the present study was to elucidate the respective importance of Chol and SM as compared to phosphatidylcholine (PC) species in the membrane-related effects of Rh2. On simple lipid monolayers, Rh2 interacted more favorably with eggSM and DOPC than with Chol and eggPC. Using Large Unilamellar Vesicles (LUVs) of binary or ternary lipid compositions, we showed that Rh2 increased vesicle size, decreased membrane fluidity and induced membrane permeability with the following preference: eggSM:eggPC > eggSM:eggPC:Chol > eggPC:Chol. On Giant Unilamellar Vesicles (GUVs), we evidenced that Rh2 generated positive curvatures in eggSM-containing GUVs and small buds followed by intra-luminal vesicles in eggSM-free GUVs. Altogether, our data indicate that eggSM promotes and accelerates membrane-related effects induced by Rh2 whereas Chol slows down and depresses these effects. This study reconsiders the theory that Chol is the only responsible for the activity of saponins.

## Introduction

Cholesterol (Chol) and sphingomyelin (SM) are essential components of mammalian plasma membranes, constituting around 35 and 25% of total lipids present in the outer plasma membrane leaflet, respectively^1^. It was for a long time believed that these lipids are randomly distributed into the plasma membrane^2^. However, studies over the last decades have suggested for the presence of transient nanometric domains enriched in Chol and SM, forming a liquid-ordered (L_o_) phase^3–5^. These highly ordered membrane domains have been proposed to serve as platforms for signaling pathways involved in cell adhesion and migration as well as cell survival and proliferation with potential implications in cancer development^6^. Altogether, this makes the lipids present in L_o_ domains an interesting target for pharmacological agents to modulate these pathways.

Among these, saponins, amphiphilic compounds widely found in plants, are attracting more and more attention based on their numerous biological activities including anticancer properties. Many of these effects seem to be related to their ability to interact and modify the plasma membrane properties, such as fluidity or permeability^7^. These membrane-related mechanisms are not completely understood yet, but the activity of some saponins such as alpha-hederin^8,9^, digitonin^10,11^ and alpha-tomatine^12,13^ have been attributed to their interaction with membrane Chol by forming complexes^14^.

**Figure 1:**
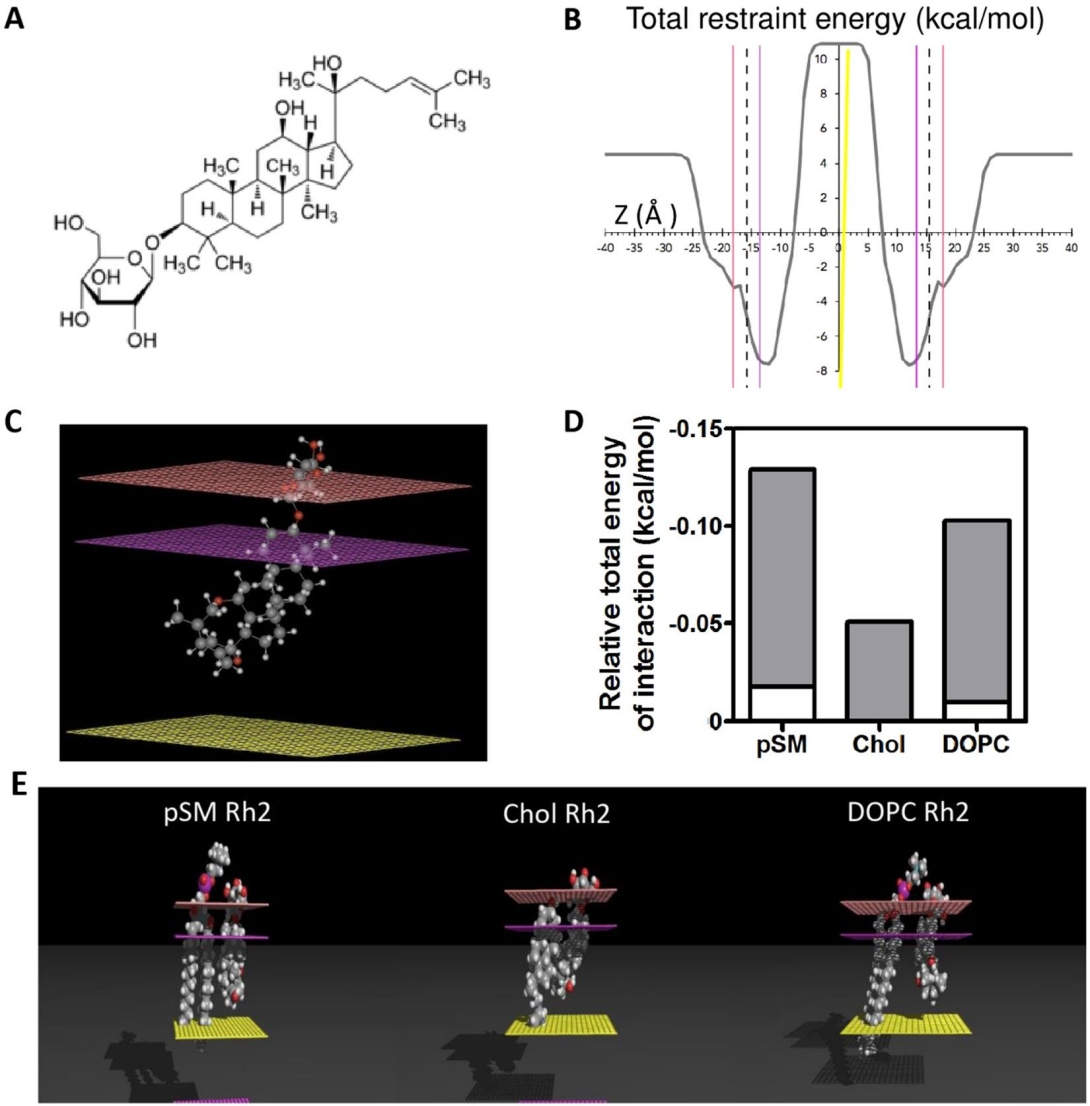
Rh2 interacts more favorably with membrane pSM and DOPC than with Chol. (**A**) Chemical structure of Rh2. (**B**,**C**) IMPALA simulation of the most favorable position of Rh2 into an implicit membrane 36 Å thick. (**B**) Energy profile of Rh2 traversing an implicit lipid bilayer. X-axis corresponds to the position of the center of mass of the saponin across the bilayer expressed in Ångström. Y-axis corresponds to total restraint energy expressed in kcal/mol. The solid vertical lines in different colors represent the different planar surfaces from the left to the right: water/membrane interface (pink), the lipid polar head/alkyl chain interface (purple), and the center of the bilayer (yellow). The vertical dotted line at ±15.75 Å corresponds to the interface between the hydrophobic and hydrophilic parts of the bilayer. (**C**) The most stable position of Rh2 into the implicit bilayer. The different planar surfaces represent the same solid vertical lines described in (**B**). Carbon atoms are dark grey, Hydrogens white, Nitrogens light blue and Oxygens red. (**D**,**E**) Interaction between Rh2 and pSM, Chol or DOPC was calculated by Hypermatrix docking method. (**D**) Relative total energy of interaction values for the interaction between a central molecule Rh2 and surrounding pSM, Chol or DOPC. Grey and white portions are the apolar and polar components of the energies, respectively. (**E**) Molecular assemblies of a Rh2 molecule interacting with a single pSM, Chol or DOPC molecule.

In this study, we focus on the ginsenoside Rh2 (structure presented in Fig. 1A), found in *Panax ginseng* and responsible to some extent for its medicinal properties and health benefits^15^. This well-known steroid saponin has been reported to disrupt L_o_ domains leading to apoptosis, via either the death receptor FAS oligomerization in human cervical cancer Hela cells^16^ or inactivation of the serine/threonine kinase Akt in human epidermoid carcinoma A431 and breast cancer MBA-MB-231 cells^16,17^. In addition, our recent publication showed that the cytotoxicity of Rh2 is accelerated by membrane Chol depletion but delayed by SM depletion in human monocytic leukemia U937 cells^18^. These observations led to the hypothesis that, contrary to membrane SM, Chol could delay the activity of ginsenoside Rh2. The aim of the present study was thus to obtain detailed knowledge about the membrane-related effects of Rh2 and to elucidate the respective importance of Chol and SM.

To these aims, we first evaluated the interaction of Rh2 with membrane *in silico* and Langmuir experiments on simple lipid monolayers composed of egg sphingomyelin (eSM), egg phosphatidylcholine (ePC), 1,2-dioleoyl-sn-glycero-3-phosphocholine (DOPC) or Chol. In a second step, we evaluated the consequences of Rh2 membrane adsorption on liposome size and a set of biophysical parameters (fluidity, fusion, lipid phase organization, shape deformation and permeability) determined on Large Unilamellar Vesicles (LUVs) or Giant Unilamellar Vesicles (GUVs) containing eSM (*i.e*. eSM:ePC, 1:1) or Chol (*i.e*. ePC:Chol, 1:1) or both of them (*i.e*. eSM:ePC:Chol, 1:1:1).

Data provided in our paper indicate that, in contrast to eSM, Chol slows down and depresses the membrane-related effects induced by Rh2. To the best of our knowledge, this study is the first to reveal the protective role of Chol in membrane-related effects induced by Rh2.

## Results

### Rh2 interacts more favorably with pSM and DOPC than Chol as revealed by molecular modeling

Firstly, molecular modeling was used to investigate the structure and molecular interactions of Rh2 with rigid 3D structures of palmitoylsphingomyelin (pSM), Chol and DOPC. The IMPALA method was used to predict the potential insertion of Rh2 into an implicit lipid bilayer^19^. The apolar aglycone moiety of Rh2 was deeply embedded into the bilayer as indicated by its most energetically stable position at ±12 Å (Fig. 1B). Interaction energies between the saponin and the aqueous medium outside of the membrane were highly positive, thus less favorable, suggesting that the most favorable state of Rh2 was within the bilayer (Fig. 1C). In addition, high energies were predicted at the bilayer center, suggesting that Rh2 would not be able to cross the bilayer. A docking method called Hypermatrix^20^ was used to calculate the energies of interaction between the central molecule, Rh2, and the surrounding lipids. These molecular assemblies showed more favorable interactions between Rh2 and pSM or DOPC in comparison to Chol molecules (Fig. 1D,E). Calculations predicted that the interactions were mainly apolar in nature (grey bars in Fig. 1D).

### Rh2 binds to lipid bilayer vesicles with the following preference: DOPC > eSM > ePC

Isothermal Titration Calorimetry (ITC) experiments were then applied to thermodynamically quantify the interaction of Rh2 with liposomes containing eSM, ePC or DOPC. Notice however that formation of liposomes with Chol was not achieved. LUVs were injected in steps into the sample cell containing Rh2 (Rh2/lipid ratio of 0.02) at 25 °C and thermodynamic parameters were determined (Table 1). The free energy of binding values (ΔG) were negative and relatively similar for the three compositions examined, indicating a favorable and spontaneous interaction. The entropy values (ΔS) were all positive, clearly indicating that the binding of Rh2 with the three types of vesicles was entropically favored but with differences depending on the lipid (eSM > DOPC > ePC). Regarding binding constants, the affinities of Rh2 with lipid bilayers occurred with the following preference: DOPC > eSM > ePC. This could be linked with the difference between DOPC and eSM or ePC liposomes in their interactions with Rh2 as revealed by the enthalpy values (∆H). For instance, the interaction of Rh2 with eSM and ePC was endothermic (∆H > 0) with higher positive enthalpic changes of eSM compared to ePC whereas Rh2 binding with DOPC was found to be exothermic (∆H < 0).

**Table 1:** Preferential binding of Rh2 to DOPC > eSM > ePC vesicles is accompanied by exo- (DOPC) or endothermic reactions (eSM, ePC).

LUVs	K (mM^−1^)	∆H (KJ.mol^−1^)	T∆S (KJ.mol^−1^)	∆G (KJ.mol^−1^)
eSM	18.03 ± 7.11	15.88 ± 3.31	49.91 ± 2.43	−34.03 ± 0.93
ePC	4.95 ± 4.65	1.06 ± 0.17	29.42 ± 4.46	−28.36 ± 4.29
DOPC	33.67 ± 1.80	−1.29 ± 0.20	34.47 ± 0.09	−35.75 ± 0.13

Thermodynamic parameters measured by isothermal titration calorimetry for the binding of Rh2 to LUVs composed of eSM, ePC or DOPC at a Rh2/lipid ratio of 0.02 at 25 °C.

### Rh2 adsorbs to a higher extent with eSM and DOPC than with Chol and ePC monolayers

**Figure 2:**
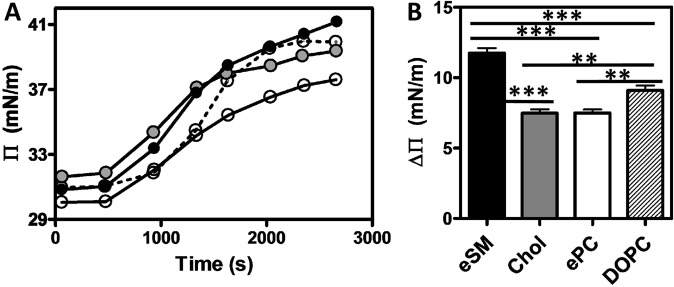
Rh2 adsorbs to a higher extent with eSM and DOPC than with Chol and ePC monolayers. Adsorption kinetics recorded at 25 °C of Rh2 on pure lipids (eSM (black), Chol (grey), ePC (white), DOPC (dotted line)). (**A**) Surface pressure – time (Π – t) curves for pure lipid monolayers at 30 ± 2 mN/m initial surface pressure recorded directly after the injection into the buffer subphase of Rh2 at the final concentration of 10 µM. (**B**) ΔΠ is the difference between the final and initial surface pressures. The curves are representative of three independent experiments and histogram data are means of these triplicated assays. One-way ANOVA with Bonferroni’s multiple comparison post test. **p < 0.01; ***p < 0.001.

To explore Rh2:Chol interaction in comparison with that of Rh2 with eSM, ePC and DOPC, we pursued our study on lipid monolayers made of these lipids by measuring adsorption kinetics via the Langmuir trough method. Lipids were spread at the water-air interface to obtain an initial surface pressure of approximately 30 ± 2 mN/m. Rh2 was then injected into the buffer subphase at the final concentration of 10 μM and its adsorption to the different films was determined by measuring the surface pressure (Π) variations as a function of time with a constant film area. The injection of Rh2 beneath the eSM, Chol, ePC or DOPC monolayers gave rise to a rapid increase of Π over time (Fig. 2A). The ΔΠ upon the adsorption of Rh2 to lipid monolayers was observed with the following preference: eSM > DOPC > ePC and Chol monolayers (Fig. 2B) in agreement with results from Hypermatrix.

### Rh2 increases eSM packing and transition temperature

**Figure 3:**
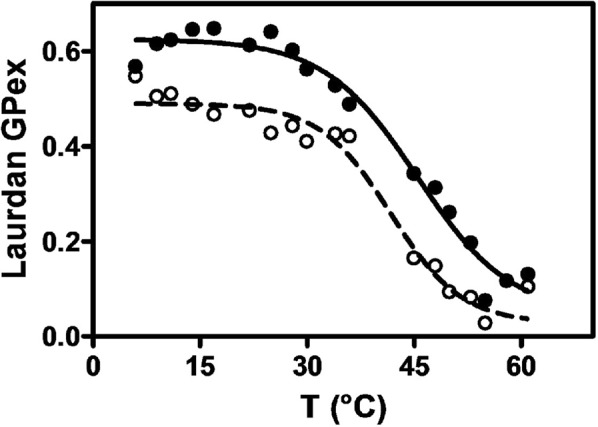
Rh2 increases eSM packing and transition temperature. Laurdan generalized polarization (GPex) was assessed at multiple temperatures from 4 to 60 °C in LUVs composed of eSM combined with Laurdan (0.5% mol) and treated for 5 min with 1% DMSO (dashed line) or at a Rh2/lipid ratio of 1 (black line). The λ_ex_ was set at 340 nm and the λ_em_ at 440 and 490 nm. The lines correspond to a non-linear regression (Hill’s function) of data values and the inflexion points correspond to the transition temperature. The curves are representative of two independent experiments.

Since Rh2 was able to highly adsorb to eSM monolayers, we then determined the effect of Rh2 on the eSM hydration and main transition temperature by evaluating the generalized polarization (GPex) of Laurdan upon increasing temperatures (Fig. 3)^21,22^. A transition temperature (T_m_) around 41 °C was observed for eSM, *i.e*. close to the values reported in the literature using differential scanning calorimetry^23,24^. A Rh2/lipid ratio of 1 increased the value of Tm to 45 °C. In addition, whatever the temperature, the GPex was higher in presence of Rh2 suggesting that the ginsenoside decreased hydration of eSM. Thus, we suggest that upon addition of Rh2, the packing order would be reinforced giving rise to lower lipid lateral diffusion coefficients. These results are in agreement with the impairment of eSM:water hydrogen-bonds and packing induced by Rh2 as suggested by ITC.

### Rh2 adsorption on mixed lipid monolayers is enhanced in the presence of eSM and the absence of Chol

**Figure 4:**
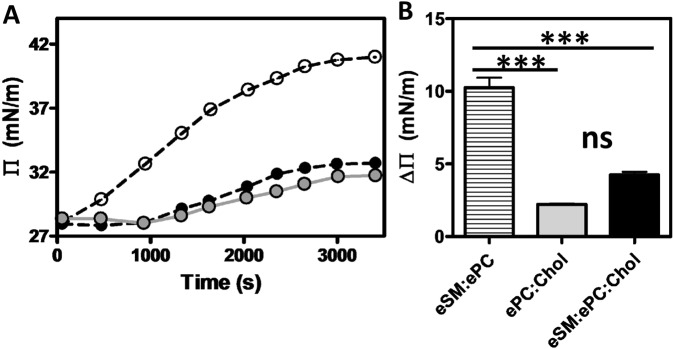
Rh2 adsorbs to a higher extent with complex lipid monolayers containing eSM but no Chol. Adsorption kinetics recorded at 25 °C of Rh2 on mixed lipids (eSM:ePC (striped bars), ePC:Chol (grey bars), eSM:ePC:Chol (black bars)). (**A**) Surface pressure – time (Π – t) curves for mixed lipid monolayers at 30 ± 2 mN/m initial surface pressure recorded directly after the injection into the buffer subphase of Rh2 at the final concentration of 10 µM. (**B**) ΔΠ is the difference between the final and initial surface pressures. The curves are representative of three independent experiments and histogram data are means of these triplicated assays. One-way ANOVA with Bonferroni’s multiple comparison post test. ns: non-significant; ***p < 0.001.

We next investigated the interaction of Rh2 with binary or ternary lipid systems. We first determined if and how Rh2 could adsorb to lipid monolayers made of either eSM:ePC (1:1), ePC:Chol (1:1) or eSM:ePC:Chol (1:1:1). Rh2 increased the Π within 500 s in eSM:ePC monolayer but only after 1400 s in monolayers containing Chol (Fig. 4A). A plateau was observed in all conditions at 2600 s but a higher ΔΠ was measured for eSM:ePC as compared to eSM:ePC:Chol and ePC:Chol monolayers (Fig. 4B). These data indicated that Rh2 adsorbed on mixed lipid monolayers with the following preference: eSM:ePC > eSM:ePC:Chol and ePC:Chol membrane.

### Rh2 increases the size of LUVs containing eSM but no Chol at a low Rh2/lipid ratio

**Figure 5:**
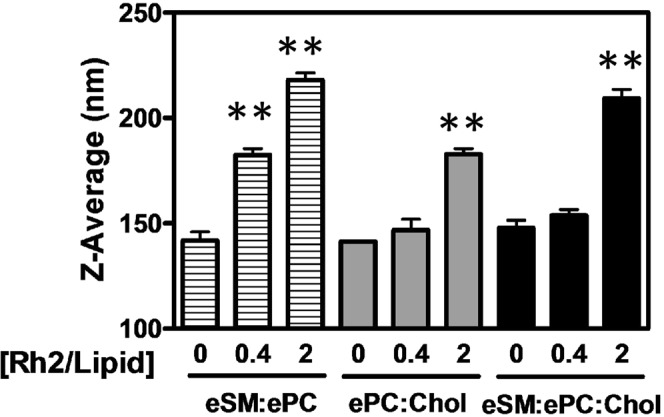
LUV size is increased at a low Rh2/lipid ratio in the presence of eSM and the absence of Chol. LUVs composed of eSM:ePC (1:1, striped bars), ePC:Chol (1:1, grey bars) or eSM:ePC:Chol (1:1:1, black bars) were incubated for 5 min with 1% DMSO or increasing Rh2/lipid ratios at 25 °C and vesicle size was determined by dynamic light scattering. Data are means of triplicated assays. One-way ANOVA with Dunnett’s post tests to compare control *versus* Rh2-treated LUVs. **p < 0.01.

Having shown the favorable adsorption of Rh2 with mixed lipid monolayers in the presence of eSM and the absence of Chol, we then turned our attention to mixed lipid bilayers as a more relevant model system. We first assessed the size of LUVs by dynamic light scattering (Fig. 5). At a Rh2/lipid ratio of 0.4, the size of eSM:ePC liposomes increased from ~140 to ~190 nm, while vesicles containing Chol remained unchanged. However, at a Rh2/lipid ratio of 2, the size of vesicles increased regardless of the liposome composition with an average vesicle diameter of ~225, ~210 and ~190 nm for eSM:ePC, eSM:ePC:Chol and ePC:Chol, respectively. These results indicated that Rh2 increased more favorably the LUV size in the presence of eSM and the absence of Chol in agreement with the preferential binding of Rh2 with monolayers containing eSM but no Chol (Fig. 4).

### Rh2 increases membrane rigidity in the presence of eSM and the absence of Chol at a low Rh2/lipid ratio

**Figure 6:**
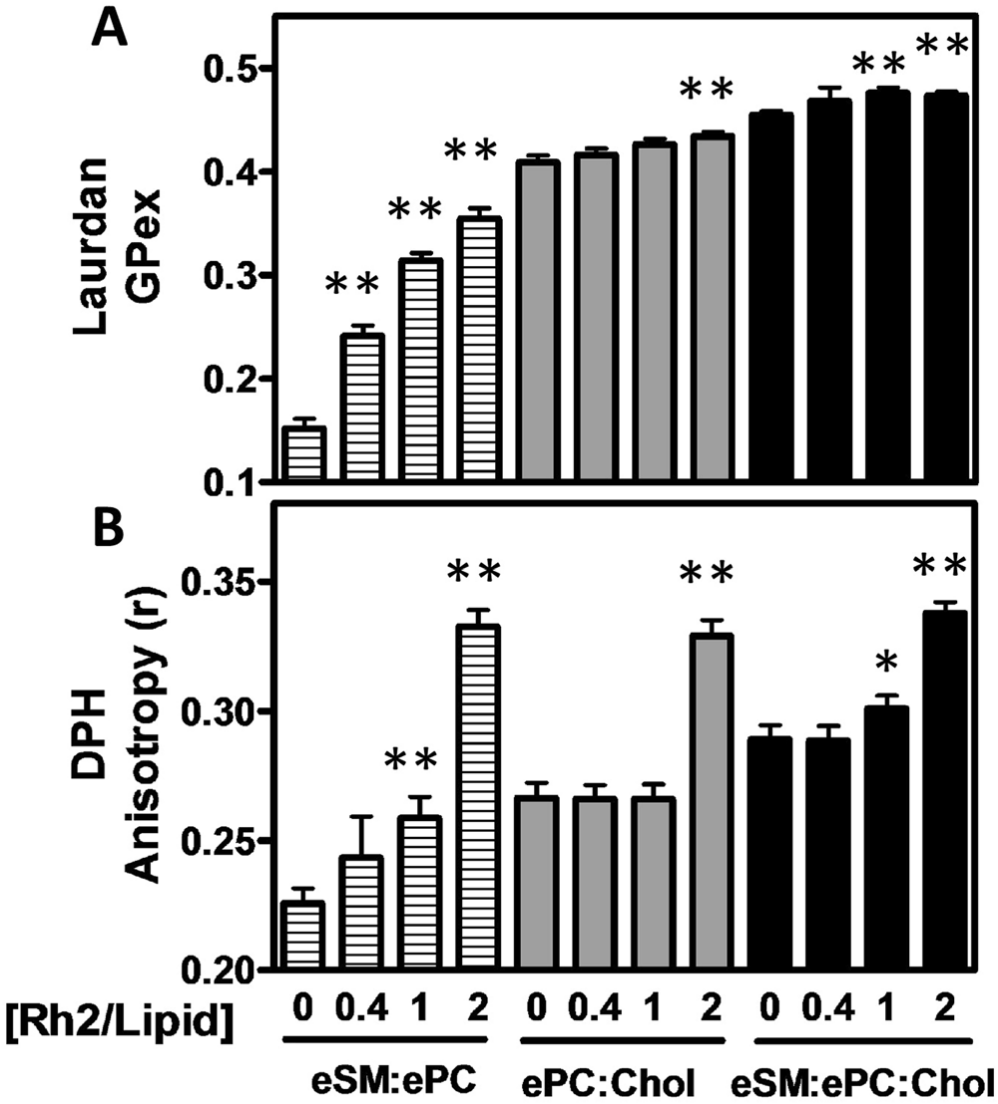
Membrane rigidity is increased at a low Rh2/lipid ratio in the presence of eSM and the absence of Chol. LUVs composed of eSM:ePC (1:1, striped bars), ePC:Chol (1:1, grey bars) or eSM:ePC:Chol (1:1:1, black bars) combined with Laurdan (0.5% mol, **A**) or DPH (0.5% mol, **B**) were incubated for 5 min with 1% DMSO or increasing Rh2/lipid ratios at 25 °C. Laurdan generalized polarization (GPex) and DPH fluorescence anisotropy (r) were measured following Rh2 treatment. The λ_ex_ was respectively set at 340 nm (Laurdan) and 365 nm (DPH) and the λ_em_ at 440 and 490 nm (Laurdan) and 425 nm (DPH). Results are means of three independent experiments in triplicates. One-way ANOVA with Dunnett’s post tests to compare control *versus* Rh2-treated LUVs. *p < 0.05; **p < 0.01.

To next determine if Rh2 could affect membrane biophysical properties to a differential extent based on membrane composition, we started by evaluating membrane fluidity by the Laurdan generalized polarization (GPex; Fig. 6A) and the DPH fluorescence anisotropy (r; Fig. 6B) on the three liposome compositions. Laurdan locates at the level of the lipid glycerol backbone and allows to detect variations in membrane phase properties through its sensitivity to the environment polarity^22^ while the DPH probe is in the hydrophobic core of the membrane and is sensitive to changes in lipid acyl chain physical properties^25^. The increase of these parameters indicates a higher rigidity in the lipid core region. In the absence of Rh2, membranes composed of eSM:ePC, ePC:Chol and eSM:ePC:Chol were characterized as the more fluid to the more rigid. After 5 min of incubation with Rh2 at a 0.4 molar ratio, GPex and anisotropy values were increased in Chol-free liposomes (eSM:ePC) but not in Chol-containing liposomes (ePC:Chol and eSM:ePC:Chol). At a Rh2/lipid molar ratio of 1, Rh2 significantly increased the eSM:ePC:Chol membrane rigidity whereas ePC:Chol exhibited no significant rigidity increase as compared to the control condition. A Rh2/lipid molar ratio of 2 was needed to increase the rigidity of the ePC:Chol system. Thus, it clearly appears that after addition of a low Rh2/lipid ratio, membrane rigidity only increased in the presence of eSM and the absence of Chol.

### Rh2 induces membrane fusion to a faster and higher extent in the absence of Chol

**Figure 7:**
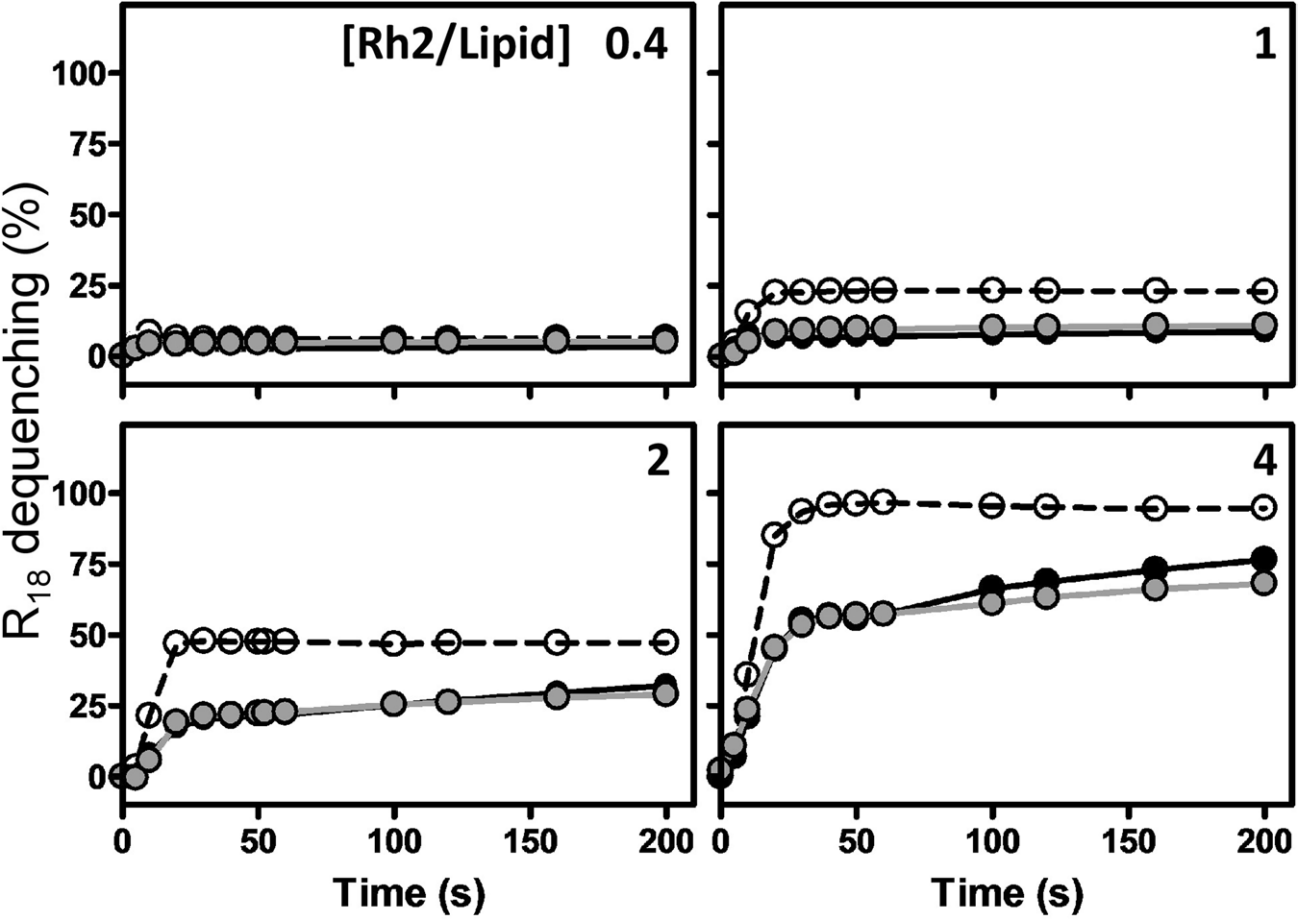
Rh2-induced membrane fusion occurs to a faster and higher extent in the absence of Chol. LUVs composed of eSM: PC (1:1, dashed lines), ePC:Chol (1:1, grey lines) or eSM:ePC:Chol (1:1:1, black lines) labeled with R_18_ were incubated with increasing Rh2/lipid ratios (indicated in the top right corner of each graph) at 25 °C. Membrane fusion of LUVs was assessed by R_18_ fluorescence dequenching. The change of fluorescence intensity was measured during 200 s, using λ_ex_ and λ_em_ at 560 nm and 590 nm, respectively. Fluorescence intensities were normalized as described in ≪Material and methods≫. The results are means of two independent experiments in triplicates.

We next evaluated the ability of Rh2 to induce membrane fusion by using the self-quenching probe R_18_ (octadecyl rhodamine B chloride). Fusion of R_18_-labeled liposomes was monitored via the increase of fluorescence over time, which results from the relief of R_18_ self-quenching due to the decrease in its surface density. At a Rh2/lipid ratio of 0.4 which increased the membrane rigidity in eSM:ePC system (Fig. 6), no R_18_ dequenching was observed (Fig. 7, ratio of 0.4). In contrast, at higher Rh2/lipid ratios, a plateau was reached after 25 s for all liposome compositions (Fig. 7, ratios of 1, 2 and 4). Importantly, Rh2 provoked faster and higher increase of R_18_ dequenching over time in Chol-free liposomes (eSM:ePC) as compared to Chol-containing liposomes (eSM:ePC:Chol and ePC:Chol). These results suggested that Rh2-induced membrane fusion to a faster and higher extent in the absence of Chol.

### Rh2 accelerates liposome permeability in the presence of eSM and the absence of Chol

**Figure 8:**
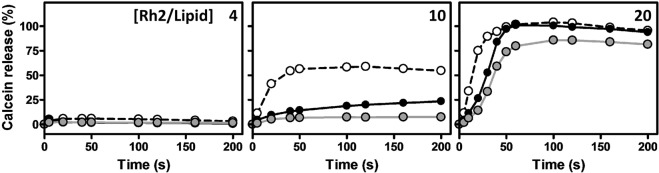
Rh2-induced membrane permeability is faster in the presence of eSM and the absence of Chol. LUVs composed of eSM:ePC (1:1, dashed lines), ePC:Chol (1:1, grey lines) or eSM:ePC:Chol (1:1:1, black lines) entrapping calcein at self-quenching concentrations were incubated with increasing Rh2/lipid ratios (indicated in the top right corner of each graph) at 25 °C. Membrane permeability was determined by the release of calcein from LUVs. The changes of fluorescence intensities were measured during 200 s, using λ_ex_ and λ_em_ at 472 nm and 512 nm, respectively. Fluorescence intensities were normalized as described in ≪Material and methods≫. Results are means of three independent experiments in triplicates.

To investigate if the insertion of Rh2 into the membrane could induce membrane permeability, the release of calcein from LUVs was measured. Membrane permeabilization is evaluated by measuring the increase of fluorescence emission of LUVs entrapping self-quenched calcein in case of release across the permeabilized membrane. At a Rh2/lipid molar ratio of 4 for which Rh2 was able to induce membrane fusion (Fig. 7), there was no calcein release (Fig. 8, ratio of 4). At a ratio of 10, the fluorescence profile of the three types of liposomes can be discriminated. For instance, after 50 s of incubation with Rh2 (at which a plateau was obtained), 50% of calcein was released from eSM:ePC, ~25% from eSM:ePC:Chol while no release was measured from ePC:Chol liposomes (Fig. 8, ratio of 10). Treatment with a Rh2/lipid ratio of 20 induced a time-dependent increase of fluorescence (Fig. 8, ratio of 20). These results showed that Rh2 accelerated membrane permeability in the presence of eSM and the absence of Chol.

### Rh2 induces positive membrane curvature in the presence of eSM and small buds followed by intra-luminal vesicles in its absence

**Figure 9:**
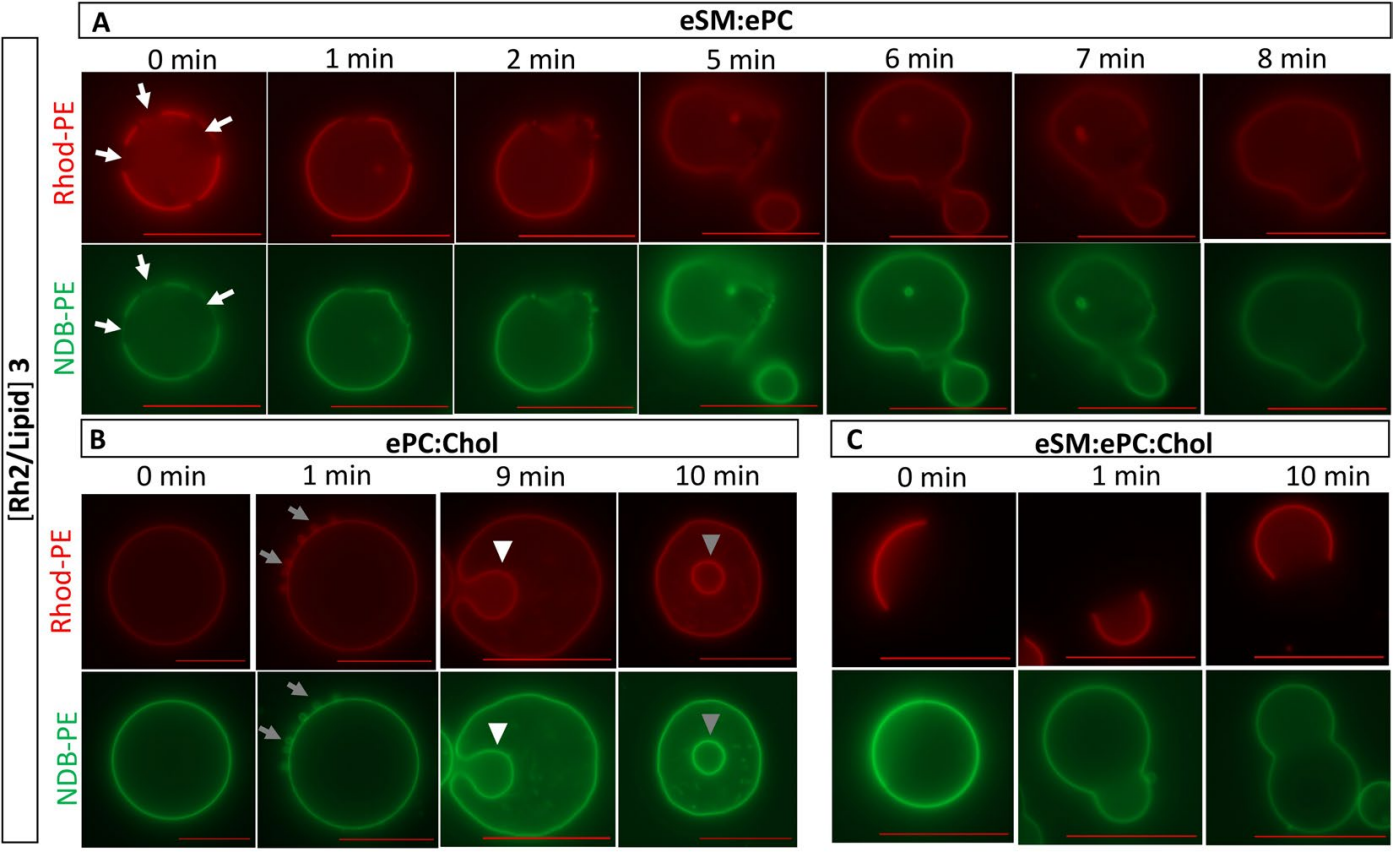
Rh2 induces positive membrane curvature in the presence of eSM and small buds followed by intra-luminal vesicles in its absence. GUVs composed of eSM:ePC (**A**), ePC:Chol (**B**) and eSM:ePC:Chol (**C**) were labeled with Rhod-PE (red) and NBD-PE (green) to reveal the liquid-disordered (L_d_) and liquid-ordered (L_o_) phases, respectively. GUVs were incubated with 1% DMSO (control, 0 min) or Rh2/lipid ratio of 3 and visualized at the indicated times at room temperature with a Zeiss wide-field fluorescence microscope (Observer.Z1) using a plan-Apochromat 40x/1.4 oil Ph3 objective. White arrows point to solid-ordered phases (S_o_), grey arrows to small buds, white arrowheads to inward membrane budding and grey arrowheads to intra-luminal vesicles. Scale bars, 20 µm. Images are representative of two independent experiments.

**Figure 10:**
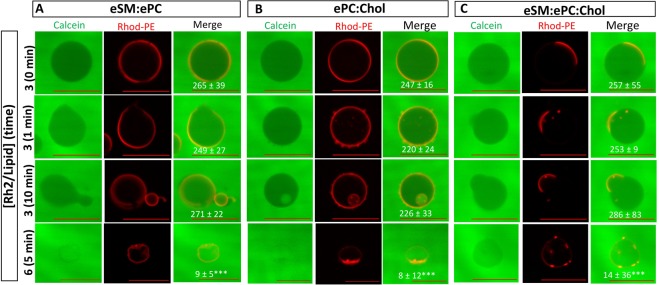
Morphology changes lead to visible membrane permeability only at a Rh2/lipid ratio of 6. GUVs composed of eSM:ePC (**A**), ePC:Chol (**B**) or eSM:ePC:Chol (**C**) were labeled with Rhod-PE (red) and diluted in 0.2 M glucose supplemented with 20 μM calcein (green). GUVs were incubated with either 1% DMSO (line 1) or with Rh2 at a Rh2/lipid ratio of 3 after 1 or 10 min (lines 2 and 3, respectively), or of 6 for 5 min (line 4) at 22 °C. The difference of green fluorescence intensities between outside and inside vesicles was measured and indicated in the merge panel. GUVs were visualized by confocal microscope with Axio Observer 1 inverted microscope equipped with a spinning disk microscope. Scale bars, 20 µm. One-way ANOVA with Bonferroni’s multiple comparison. ***p < 0.001. Images are representative of two independent experiments and at least 15 GUVs were analyzed per experiment.

We then took benefit from the resemblance of GUVs with cell membranes in terms of size and membrane curvature to visualize if Rh2 could change the GUV morphology. To this aim, GUVs were labeled with 1,2-dioleoyl-sn-glycero-3-phosphoethanolamine-N-(lissamine rhodamine B sulfonyl) (Rhod-PE, red channel) and N-(7-Nitrobenz-2-Oxa-1,3-Diazol-4-yl)-1,2-Dihexadecanoyl-sn-Glycero-3-Phosphoethanolamine (NBD-PE, green channel) to probe the liquid-disordered (L_d_) and liquid-ordered (L_o_) phases, respectively^26^. The time course of morphology changes was studied by incubating GUV suspensions with a fixed Rh2/lipid ratio of 3, for which membrane fusion but not permeability occurred (see above results). Control GUVs exhibited a spherical shape with no detectable morphology changes for the whole duration of the experiment (data not shown). In control eSM:ePC GUVs, solid-ordered phases (S_o_) were observed (dark areas, white arrows at Fig. 9A, 0 min), as expected^27,28^. Following the same GUV upon addition of Rh2, the progressive formation of positive membrane curvature was accompanied by the progressive vesicle budding after 1 and 2 min (Fig. 9A, 1 and 2 min). Then, we were able to evidence membrane fusion and fission from 5 to 8 min (Fig. 9A, 5 to 8 min). In ePC:Chol GUVs, no phase separation was observed in the absence of Rh2 (Fig. 9B, 0 min). After 1 min, Rh2 promoted the formation of small buds (grey arrows) around the membrane that remained connected to the parent GUV, consistent with the induction of membrane curvature changes (Fig. 9B, 1 min). After 9 min, the small buds progressively disappeared but inward budding of GUV membranes appeared (white arrowheads at Fig. 9B, 9 min), leading to intra-luminal vesicles (ILVs) after 10 min (grey arrowheads at Fig. 9B, 10 min). In eSM:ePC:Chol, lipid phase separation was observed, as expected^23,28^ (Fig. 9C, 0 min). Over the course of Rh2 treatment, positive membrane curvature occurred and L_d_ domain preferentially localized into these highly curved domains (Fig. 9C, 1 and 10 min). These results suggested that the insertion of Rh2 into the membrane promoted positive membrane curvature in the presence of eSM and the formation of small buds followed by ILV formation in the absence of eSM.

### Rh2-induced morphology changes are not directly associated with membrane permeabilization

To determine the potential link between GUV morphology changes and Rh2-induced membrane permeability, GUVs were placed in IBIDI chambers filled with glucose and calcein and were observed by confocal microscopy. Control GUV membranes containing sucrose were impermeable to free calcein present in the solution, showing a difference of fluorescence intensity between outside and inside of the GUVs (values noted in the merge panels; Fig. 10 A–C; line 1). This difference was maintained for the whole duration of the incubation with DMSO (data not shown). At a Rh2/lipid ratio of 3 and in the presence of eSM, the membrane budding induced by Rh2 reported in Fig. 9A,C was clearly visible but did not allow the entry of calcein into the GUVs (Fig. 10A,C; lines 2 and 3). In ePC:Chol GUVs, the formation of calcein-free small buds mediated by outward budding of GUV membranes was observed already after 1 min (Fig. 10B; line 2) while the formation of ILVs was noticed after 10 min (Fig. 10B; line 3). The difference of fluorescence intensity between outside the GUVs and inside those ILVs was 80 ± 36 which was lower than the difference between outside and inside control GUVs. This indicated that the calcein contained in the solution was encapsulated inside small vesicles during their formation, consistent with the formation of calcein-containing ILVs through inward indentation of the limiting GUV membrane^29^. Thus, Rh2-induced morphology changes were not directly associated with membrane permeability. A Rh2/lipid ratio of 6 was needed to induce wrinkled borders and entry of calcein through the membrane of the three GUV compositions, leading to the suppression of difference of fluorescence intensities between outside and inside of GUVs (Fig. 10A–C; line 4). The latter results are compatible with the creation of membrane pores by Rh2 whatever the GUV composition.

### In contrast to Rh2, digitonin modulates membrane biophysical properties in a Chol-dependent manner

**Figure 11:**
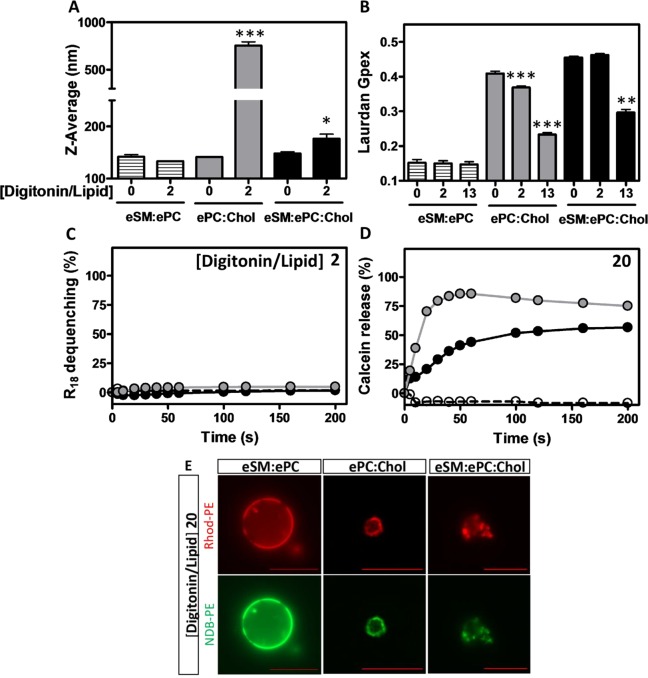
Digitonin changes membrane biophysical properties in a Chol-dependent manner. (**A**–**D**) LUVs composed of eSM:ePC (1:1, striped bars or dashed lines), ePC:Chol (1:1, grey bars or lines) or eSM:ePC:Chol (1:1:1, black bars or lines) were incubated with 1% DMSO or increasing digitonin/lipid ratios as indicated at 25 °C. (**A**) LUV size was determined by dynamic light scattering. Unpaired t-test was used to compare control *versus* digitonin-treated LUVs. (**B**) Fluidity was determined by GPex values on LUVs labeled with Laurdan. One-way ANOVA with Dunnett’s post tests to compare control *versus* digitonin-treated LUVs. *p < 0.05; **p < 0.01; ***p < 0.001. (**C**,**D**) Membrane fusion and permeability were assessed by R_18_ fluorescence dequenching (**C**) and calcein self-quenching release (**D**), respectively. All the results are means of two independent experiments in triplicates. (**E**) GUVs composed of eSM:ePC, ePC:Chol or eSM:ePC:Chol were labeled with Rhod-PE (red) and NBD-PE (green) and were incubated with a digitonin/lipid ratio of 20 at room temperature. After 2 min following digitonin treatment, pictures were taken with a Zeiss wide-field fluorescence microscope (Observer.Z1) using a plan-Apochromat 40x/1.4 oil Ph3 objective. Scale bars, 20 μm. Images are representative of two independent experiments.

Altogether, the data reported above indicated that Rh2 modified to a higher extent biophysical properties of LUVs in the presence of SM and the absence of Chol. Since those results challenged the usual view that some saponins mediate their membrane effects through interaction with Chol, the same series of experiments were carried out with digitonin, a steroid saponin known to require Chol for its membrane-related effects^11^. Using the same three liposome compositions, we evaluated the effects of digitonin on vesicle size (dynamic light scattering, Fig. 11A), membrane fluidity (Laurdan, Fig. 11B), fusion (R_18_ dequenching, Fig. 11C) and permeability (calcein release, Fig. 11D). The results clearly showed that treatment with digitonin increased vesicle size, membrane fluidity and permeability only in Chol-containing systems without any effect on membrane fusion even at a Rh2/lipid ratio of 13 (data not shown). Interestingly, ePC:Chol vesicles were more susceptible to digitonin as compared to eSM:ePC:Chol, suggesting an influence of Chol concentration. Consistently, digitonin rapidly disrupted the GUV membrane in the presence of Chol but not in its absence (Fig. 11E) even after long time (2 hours, data not shown). Altogether, these results supported the essential role of Chol in the membrane-related effects induced by digitonin and corroborated our observations that Rh2 interacted with the membrane in a differential way.

## Discussion

The membrane-related effects of saponins are often considered as Chol-dependent. Our recent observations with the saponin ginsenoside Rh2 let us to reconsider this purpose and to propose that, in contrast to eSM, membrane Chol could decrease the activity of Rh2^18^. We here tested this hypothesis by determining Rh2:membrane interaction by *in silico* experiments as well as *in vitro* studies on lipid monolayers and bilayers while modulating the lipid composition, *i.e*. the presence or absence of Chol or eSM. *In silico* studies clearly revealed higher relative interaction energy between Rh2 and pSM or DOPC in comparison to Chol, which could result from higher hydrophobic and van der Waals forces but also higher hydrogen bond energies. Accordingly, studies on lipid monolayers showed that Rh2 adsorbed to a higher extent to eSM or DOPC in comparison to Chol and ePC monolayers.

As revealed by ITC, interactions between Rh2 and both eSM and DOPC are favorable with negative values for variation of free energy. They differ however since interaction with eSM was endothermic and mainly driven by entropy whereas interaction with DOPC was slightly exothermic with lower values for variations of entropy. Regarding the interaction of Rh2 with ePC and DOPC, the binding affinity between Rh2 and ePC was lower than with DOPC vesicles which could indicate that variations in the fatty acyl chain lengths, the number of unsaturation found in ePC composition (containing around 30% DOPC) and the resulting biophysical membrane properties could reduce membrane binding of Rh2.

To be closer to physiological condition, we then turned to mixed lipid bilayers. Even if biological membranes are more complex than artificial lipid bilayers (due to the presence of membrane and cytoskeletal proteins but also of membrane asymmetry), our data are consistent with those previously obtained in human cancer cell lines (A549, THP-1, and U937) which highlighted that membrane Chol depletion accelerates Rh2-induced apoptosis^18^. It can be deduced that Rh2 did not specifically interact with Chol which could instead delay and/or decrease the Rh2-induced membrane-related effects. Several hypotheses can be proposed to explain such a delayed/decreased effect. First, due to space constriction, Rh2 could have to at first remove Chol from the membrane, a mechanism reported for digitonin which extracts Chol and forms a complex with the latter^30^. However, three lines of evidence reported here do not support this possibility. Firstly, digitonin increased the size and induced membrane lysis only in Chol-containing liposomes, as previously observed by Sudji *et al*.^11^, whereas the action of Rh2 was instead delayed and depressed by the presence of membrane Chol. Secondly, Rh2 was able to modulate membrane biophysical properties even without Chol or eSM. Thirdly, based on GPex value of Laurdan, we observed that digitonin increased membrane fluidity whereas Rh2 decreased it, suggesting that Rh2 could preferentially stay into the membrane. To reinforce this idea, hydroxypropyl-β-cyclodextrin (HPβCD), also known to capture Chol from the membrane, has been shown to decrease the surface pressure (Π) of Chol monolayers^31^, in contrast to Rh2. Moreover, like digitonin, alpha-hederin increases membrane fluidity and membrane permeability due to the formation of saponin:Chol complexes. The differential effect between Rh2 and alpha-hederin could be related to the less profound insertion into the membrane hydrophobic core of Rh2 than alpha-hederin owing to two sugar chains, thereby increasing the surface of interaction with Chol and preventing polar interactions between Chol and phospholipids^32^. This highlights the importance of saponin nature since other saponins than Rh2, including chikusetsusaponin III or oleanolic acid glycosylated at both C-3 and C-28, induce Chol-independent membrane disruption^33,34^.

A second hypothesis for explaining the protective role of Chol is through its effect on membrane rigidity, decreasing the possibility for Rh2 to adsorb and interact with the membrane. In agreement with this hypothesis, eSM:ePC liposomes were more fluid than liposomes containing Chol and were also the most sensitive to Rh2. However, even if ePC:Chol and eSM:ePC:Chol liposomes were classified from the more fluid to the more rigid, ePC:Chol vesicles were less susceptible to the action of Rh2 as compared to eSM:ePC:Chol vesicles.

Third, the delayed/depressed effect of Chol could also be explained through its favorable interaction with SM in lipid domains and its similarity with Rh2 structure. Chol could compete with Rh2, thereby depressing Rh2:SM interaction and related effects. Such interaction between Rh2 and lipid clusters is in agreement with previous studies showing the disruption of lipid domains by Rh2^16,17^. Rh2 insertion could in turn increase membrane rigidity and line tension, resulting into the formation of positive membrane curvature to finally reduce the line tension^35^. Accordingly, using coarse-grained molecular dynamics simulations, Lin and Wang have shown that the interaction of dioscin saponin with membrane Chol leads to the destabilization of the L_o_-phase and the formation of positive membrane curvature which may eventually result in the hemolysis of red blood cells^36^.

The favorable interaction of Rh2 with eSM monolayer is reminiscent to the specific binding to membrane SM of pore-forming proteins actinoporins (*e.g*. equinatoxin and sticholysin)^37,38^. Hence, binding of the toxins and their permeabilization ability only occur in model systems exhibiting phase coexistence (L_o_ and L_d_)^39^. Based on our data related to GUV phase separation and LUV membrane permeability, we rule out a similar mode of action for Rh2. For instance, whatever the presence and the type of GUV phase boundaries, Rh2 induced alterations of GUV morphology and membrane permeability.

In conclusion, our work challenged the usual view that most saponins mediate their membrane-related effects through an interaction with Chol. Here the activity of Rh2 is enhanced by the interaction with membrane SM but depressed by Chol. Further studies are required to determine the structure-activity relationship between different saponins and eventually predict the mechanisms of their interactions with membrane.

## Materials and Methods

### Materials

Ginsenoside Rh2, calcein, Sephadex® G-50, LH-20 and 1,6-diphenyl-1,3,5-hexatriene (DPH) were purchased from Sigma-Aldrich (St. Louis, MO, USA). Ginsenoside Rh2 (chemical purity ≥97.0%) was dissolved in 100% ethanol. Rh2 residue was evaporated and resolubilized in buffer solution (10 mM Tris-HCl, 159 mM NaCl pH 7.4, containing 1% DMSO). Calcein was dissolved in 6 N NaOH and subjected to size-exclusion chromatography through a Sephadex® LH-20 column. The L-α-phosphatidylcholine (ePC – Egg, Chicken), sphingomyelin (eSM – Egg, Chicken), cholesterol (Chol - Ovine Wool), 1,2-dioleoyl-*sn*-glycero-3-phosphocholine (DOPC) and 1,2-dioleoyl-*sn*-glycero-3-phosphoethanolamine-*N*-(lissamine rhodamine B sulfonyl) (ammonium salt) (Rhod-PE) were purchased from Avanti Polar Lipids Inc. (Alabaster, AL, USA). *N*-(7-Nitrobenz-2-Oxa-1,3-Diazol-4-yl)-1,2-Dihexadecanoyl-*sn*-Glycero-3-Phosphoethanolamine, Triethylammonium Salt) (NBD-PE) were ordered by Life technologies (Leusden, Netherlands). 6-Dodecanoyl-2-Dimethylaminonaphthalene (Laurdan) and Octadecyl Rhodamine B Chloride (R_18_) were acquired from Thermo scientific (Rockford, IL USA). Lipids and lipid probes were dissolved in CHCl_3_, except DPH, which was dissolved in tetrahydrofuran (THF), and were kept at −20 °C. All organic solvents used were from VWR (Radnor, PA, USA).

### *In silico* methods

IMPALA and Hypermatrix methods have been previously described in details^40^.

### Adsorption kinetics

The adsorption kinetics of Rh2 into lipid monolayer was measured by following changes in the surface pressure. Experiments were assayed in an automated Langmuir trough (KSV Mini-trough KSV instruments Ltd., Helsinki, Finland- width = 7.5 cm, length = 20 cm) with two hydrophophilic Delrin mobile barriers, a platinum Wilhelmy plate and a temperature probe. The system was enclosed in a Plexiglas box, and the temperature was maintained at 25 ± 1 °C. Lipid mixed solutions were dissolved at a concentration of 1 mM in CHCl_3_ and were added at the air-water interface with a micro-syringe (Hamilton, USA) until surface pressure (∏) reached 30 ± 2 mN/m. After reaching the equilibrium, ginsenoside Rh2 was carefully injected into the 80 ml subphase (buffer 10 mM Tris-HCl, 159 mM NaCl pH 7.4) beneath the lipid monolayer. The subphase is stirred with magnetic stirrer using specialized injection supports. The variation of the surface pressure was recorded in function of time immediately after the injection of Rh2 until a plateau was observed.

### Preparation and analysis of Large Unilamellar Vesicles

LUVs composed of eSM:ePC (1:1), eSM:ePC:Chol (1:1:1) or ePC:Chol (1:1) (molar ratio) were prepared by the extrusion from Multilamellar Lamellar Vesicles (MLVs)^41^. In brief, lipids were diluted CHCl_3_ at the desired molar ratio. The organic solvent was then removed by evaporation in rotary evaporator (Buchi Rotavapor R-200, Switzerland). The residual lipid films after drying under vacuum overnight were hydrated with buffer solution (10 mM Tris-HCl, 135 mM NaCl pH 7.4). This suspension was submitted to five cycles of freezing/thawing. The latter were extruded through a polycarbonate Nuclepore Track Etch membrane filter (100 nm pore size filter, 19 times) using a mini-extruder system (Avanti Polar Lipids) to form LUVs. The quality control of LUVs was determined by the polydispersity index (PDI) (PDI < 0.2) and particle size distribution (z-average ± 150 nm) via dynamic light scattering (see below). Total phospholipids were determined by phosphorus assay and diluted to the desired final concentration in the buffer solution^42^.

### Isothermal Titration Calorimetry

Isothermal Titration Calorimetry techniques were assessed using a VP-ITC (MicroCal Northampton, MA, USA) to provide a complete thermodynamic description of the interaction between Rh2 and LUVs made with eSM, ePC and DOPC. All solutions were prepared with the same buffer constituted of 10 mM Tris-HCl, 159 mM NaCl pH 7.4. Prior each analysis, all solutions were degassed using sonicator bath. The cell (volume 1.4045 ml) for the ITC measurement was filled with Rh2 solution at a concentration of 40 μM and stirred at a speed of 305 rpm. The reference cell is filled with milliQ water Titration was carried out at 25 °C using 300 μl syringe filled with the LUV suspension at 2 mM and series of injections of 10 μl were made with a delay of 360 s between each succession injection to allow steady state to be attained. Data were processed by software ORIGIN 7 (Originlab, Northampton, MA, USA) using the cumulative model as described in Razafindralambo *et al*.^43^.

### Size of Large Unilamellar Vesicles

The average diameter of liposomes was determined in a 12 mm square polystyrene cuvette containing LUV suspension by dynamic light scattering (DLS) technique at 25 °C using a Zetasizer Nano SZ equipment (Malvern Instruments Ltd, UK) equipped with a helium-neon laser and added back scattering detection at 173°.

### Liposomal membrane fluidity and transition temperature

The effect of Rh2 on liposomal membrane fluidity was determined by measuring the values of generalized polarization (GPex) of Laurdan and the fluorescence anisotropy of DPH^25,44^ at 25 °C. The solution of Laurdan or DPH were added at 0.5% mol to the mixed lipid suspension before the evaporation. GPex of Laurdan was measured from the equation (1).

1 $${{\rm{GP}}}_{{\rm{ex}}}={({\rm{I}}}_{{\rm{440}}}\,-\,{{\rm{I}}}_{{\rm{490}}})/{({\rm{I}}}_{{\rm{440}}}+{{\rm{I}}}_{{\rm{490}}})$$

where I_440_ and I_490_ are the λ_em_ at 440 and 490 nm, respectively recorded with the λ_ex_ at 340 nm. To determine transition temperature of eSM liposomes, Laurdan generalized polarization was assessed at multiple temperatures from 4 to 60 °C.

The fluorescence anisotropy value (r) of DPH was determined via the equation (2):

2 $$({\rm{r}})=({{\rm{I}}}_{{\rm{vv}}}\,-\,{\rm{G}}{{\rm{I}}}_{{\rm{vh}}})/{({\rm{I}}}_{{\rm{vv}}}+{\rm{2}}{\rm{.G}}/{{\rm{I}}}_{{\rm{vh}}})$$

where I_vv_, and I_vh_ are the fluorescence intensities with the excitation and emission polarization filters in vertical (v) and horizontal (h) orientations, respectively. The G factor is an inherent factor to the spectrophotometer used. The fluorescence polarization was measured at λ_ex_ and λ_em_ of 365 nm and 425 nm, respectively.

### Membrane permeability of Large Unilamellar Vesicles

The leakage from LUVs of entrapped calcein at self-quenching concentration was followed to determine the effect of Rh2 on membrane permeability^45^. Briefly, the dried lipid film was hydrated with a solution of 10 mM Tris-HCl, 135 mM NaCl pH 7.4 containing purified calcein (73 mM). LUVs were then eluted through a Sephadex-G50 column to remove the non-encapsulated calcein. Fluorescence intensities were continuously recorded during 200 s at 25 °C after the addition of increasing concentrations of Rh2 in the liposome suspension. The λ_ex_ and λ_em_ were 472 and 512 nm, respectively. The percentage of calcein release from liposomes was calculated using the equation (3):

3 $$ \% {\rm{release}}={[({\rm{F}}}_{{\rm{t}}}\,-\,{{\rm{F}}}_{{\rm{ctrl}}})/{({\rm{F}}}_{{\rm{tot}}}\,\mbox{--}\,{{\rm{F}}}_{{\rm{ctrl}}})]\,\ast \,{\rm{100}}$$

where F_t_ represents the fluorescence intensity at the time t following the treatment with Rh2. F_ctrl_ corresponds to the baseline fluorescence of liposomes at the same time and F_tot_, maximum fluorescence is the fluorescence measured after the addition of 0.1% Triton X-100 to induce maximum release.

### Membrane fusion of Large Unilamellar Vesicles

Lipid mixing assay is based on self-quenching of R_18_ (octadecyl rhodamine B chloride) in LUVs^46^. The R_18_ hydrophic probe was incorporated at 5.7% mol into a lipid mixture before its evaporation. R_18_ labeled liposomes were mixed at a ratio of 1:4 with unlabeled liposomes adjusted to the same concentration. The changes of fluorescence intensity were measured during 200 s at 25 °C, using λ_ex_ and λ_em_ at 560 nm and 590 nm, respectively. The percentage of liposomal fusion was calculated using the equation (3).

### Preparation and analysis of Giant Unilamellar Vesicles

GUVs were prepared using the electroformation method^47^. To investigate phase separation, mixtures of eSM:ePC (1:1), eSM:ePC:Chol (1:1:1) or ePC:Chol (1:1) combined with 0.2% mol Rhod-PE and 0.5% mol NBD-PE were prepared. A small volume of lipid solution was spread and dried inside the chamber of 1 mm thick silicon gasket placed on the surface of an ITO coated glass lamella. The electroformation chamber was filled with 0.2 M sucrose and overlaid with a second ITO-coated slide. The GUVs were formed by exposure to a sinusoidal alternating current of 10 Hz and 1 V for 2 h at 60 °C. The volume containing GUVs were collected from the slide and lipid concentration was determined based on a calibration curve of NBD-PE fluorescence intensity. GUVs were diluted in 0.2 M glucose and placed in a μ-Slide 8-well chamber from IBIDI (Martinsried, Germany) and observed in the red channel for Rhod-PE (λ_ex_, 561 nm and λ_em_, 617 nm) and in the green channel for NDB-PE (λ_ex_, 488 nm and λ_em_, 530 nm) at room temperature. To investigative the permeabilizing effect of Rh2, same GUV compositions combined with 0.2% mol Rhod-PE were diluted in 0.2 M glucose supplemented with 20 μM calcein and the difference of fluorescence intensity between outside and inside vesicle was measured. GUVs were visualized either with a Zeiss wide-field fluorescence microscope (Observer.Z1) using a plan-Apochromat 40x/1.4 oil Ph3 objective or a Axio Observer 1 inverted microscope equipped with a model CSU-X1 Yokogawa spinning disk and Plan-Apochromat 40x/1.40 oil DIC. Images were analyzed with the Carl Zeiss AxioVision 4.8.2 software.

### Fluorescence spectroscopy measurement

All fluorescence measurements were carried out with a LS55 luminescence spectrometer from Perkin Elmer (Waltham, MA, USA). The spectrophotometer was equipped with a temperature-controlled sample holder being set at 25 °C.

## Data Availability

Data are expressed as means ± SEM. All statistical analyses were performed with the GraphPad Prism 4.03 software (GraphPad software, San Diego, CA, USA).
